# Diversity of Non-Biting Midge Larvae Assemblages in the Jacuí River Basin, Brazil

**DOI:** 10.1673/031.012.12101

**Published:** 2012-10-21

**Authors:** Elzira Cecília Serafini Floss, Carla Bender Kotzian, Márcia Regina Spies, Elisangela Secretti

**Affiliations:** ^1^ Programa de Pós-Graduação em Biodiversidade Animal, Centro de Ciências Naturais e Exatas, Universidade Federal de Santa Maria. Faixa de Camobi km 9, 97105-900, Santa Maria, RS, Brazil; ^2^ Departamento de Biologia e PPG Biodiversidade Animal, Centro de Ciências Naturais e Exatas, Universidade Federal de Santa Maria. Faixa de Camobi km 9, 97105-900, Santa Maria, RS, Brazil; ^3^ Universidade Federal do Pampa (Unipampa - Campus São Gabriel) Av. Antônio Trilha 1847, São Gabriel, RS. CEP 97300-000, Brazil; ^4^ Curso de Graduação em Ciências Biológicas, Centro de Ciências Naturais e Exatas, Universidade Federal de Santa Maria. Faixa de Camobi km 9, 97105-900, Santa Maria, RS, Brazil

**Keywords:** Chironomidae, environmental variables, inventory, Neotropics, regional scale

## Abstract

The richness and composition of a mountain-river chironomid larvae assemblage in the Jacuí River basin, Brazil were studied, and compared with other riverine non-biting midge larvae assemblages previously studied in the country. Additionally, the influence of some regional-scale environmental characteristics on the spatial distribution of these assemblages was tested. The specimens were collected at 12 sites in the middle course of the Jacuí River basin (in the state of Rio Grande do Sul) between April 2000 and May 2002. Around 100 taxa were recorded. The dominant taxa belonged to the genera *Rheotanytarsus, Cricotopus, Polypedilum*, and *Pseudochironomus*. Twenty-two rare taxa were found, representing 22% of the total of taxa inventoried. Fourteen genera (*Aedokritus, Axarus, Endotribelos, Kiefferulus, Manoa, Oukuriella, Phaenopsectra, Stenochironomus, Xenochironomus, Xestochironomus, Cardiocladius, Metriocnemus, Paracladius*, and *Rheocricotopus*) represent new occurrences in Rio Grande do Sul. The similarity analysis of the chironomid larvae assemblages inventoried in 32 regions of Brazil indicated five groups with similarity higher than 50%. The groups, when the effects of spatial autocorrelation were removed, displayed a weak positive correlation between the assemblage composition and the aquatic system or hydraulic conditions and the hydrographic basin, and a weak negative correlation in relation to the biome. The altitude showed no correlation with the composition of the assemblage. The relatively high richness of the region surveyed in relation to other Brazilian regions corroborates some tendencies already noted in other parts of the world, such as: i) lotic systems may constitute an exception to the rule that diversity is greater in tropical regions, ii) regions of transitional relief may contain the greatest richness of Chironomidae, and iii) in rivers, the group might have its spatial distribution influenced to a greater extent by local environmental characteristics than by regional ones.

## Introduction

The family Chironomidae is one of the most diversified aquatic insect groups, occurring in all zoogeographical regions, including Antarctica (Coffman 1995). On a global scale, 4147 species with an obligatory aquatic phase are known, attributed to 339 genera (Ferrington 2008), and grouped in 22 tribes and 11 subfamilies (Epler 2001). However, the true diversity must be still higher; estimates suggest that 8000 to 20,000 species should occur (Coffman 1995).

The non-biting midges are important inhabitants of freshwater aquatic ecosystems, where their larvae reach high densities (Trivinho-Strixino and Strixino 1999; Trivinho-Strixino 2011). They constitute an important item in the trophic chain, representing the main food of many fish and birds (Porinchu and MacDonald 2003; Sánchez et al. 2006; Fagundes et al. 2007). Additionally, they are an important tool in ecological (Armitage et al. 1995) and paleoecological (Walker 1998) studies, as well as in environmental evaluation (Rosenberg 1992), agricultural entomology (Ferrarese 1993), and public-health research (Cranston 1995a).

The chironomids are important elements of the faunas of lacustrine and fluvial biotopes (Trivinho-Strixino and Strixino 1999). They also occur in reservoirs, although in lower richness and abundance (McKie et al. 2005; Jacobsen et al. 1997), and with different species composition (Rossaro et al. 2006). Studies analyzing the environmental factors that influence the spatial distribution of their assemblages are being developed. Landscape factors such as altitude, area size, order of river segments, drainage basin, phytogeographical unit, riparian vegetation, environmental preservation of the area, temperature, and hydraulic conditions (lentic/lotic) (e.g., Rossaro 1991; Jacobsen et al. 1997; Kleine and Trivinho-Strixino 2005; McKie et al. 2005; Morrone 2006; Rossaro et al. 2006; Roque et al. 2007; Luoto 2009; Marziali et al. 2009; Sonoda et al. 2009; Roque et al. 2010) are mentioned among the variables affecting assemblage distributions. However, studies conducted at larger spatial scales, with a geographical or regional (*sensu* Sandin and Johnson 2004) approach, are rare.

The Neotropical region, third in number of species of Chironomidae, harbors 618 species comprising 154 genera (Ferrington 2008). However, the diversity of the family is far from being well known. In Brazil, only 354 species have been described, but estimates suggest that there are about 1500 in total (Trivinho-Strixino 2011). Additionally, inventories and ecological studies about the assemblages of Chironomidae have concentrated on the northern and southeastern regions of the country (ie.g., Callisto and Esteves 1998; Sanseverino et al. 1998; Serrano et al. 1998; Henriques-Oliveira et al. 1999; Siqueira and Trivinho-Strixino 2005; Sanseverino and Nessimian 2007; Roque et al. 2010). In the southern region, which has a climate tending toward temperate (Maluf 2000), studies are incipient, and focus primarily on lentic environments such as lakes and wetlands (e.g., Wiedenbrug et al. 1997; Stenert et al. 2004; Panatta et al. 2006, 2007). The few studies on the spatial distribution of the assemblages of larvae of Neotropical Chironomidae have focused mainly on local or small regional scales (e.g., Corbi and Trivinho-Strixino 2008a; Sonoda et al. 2009).

The middle course of the Jacuí River (southern Brazil) contains a very diversified macroinvertebrate community, as evidenced during the environmental program “Monitoring of mollusk vectors of human diseases” conducted for the construction of the Dona Francisca Hydroelectric Power Station (see Neri et al. 2005 for Heteroptera; Spies et al. 2006 for Trichoptera; Siegloch et al. 2008 for Ephemeroptera; and Pires 2011 for Odonata). Chironomidae was the most abundant family found in the river. Thus, this study presents the first survey on the diversity of a non-biting midge assemblage in a temperate climate, of a montane river in southernmost Brazil. Additionally, the similarity between this assemblage and other chironomid larvae assemblages previously studied in Brazilian rivers was also analyzed, testing the influence of regional-scale environmental factors such as altitude, biome, hydrographic basin, and hydraulic conditions, on the spatial distribution patterns of chironomid larvae assemblages, by means of four hypothesis matrices.

## Materials and Methods

### Study area

The study was carried out in the Jacuí River basin, in the state of Rio Grande do Sul, southern Brazil. Its main headwaters are in the Planalto (central plateau) region, at a mean altitude of approximately 730 meters above sea level, and its mouth is in the Central Depression, where it contributes to formation of the Jacuí delta, in Guaíba Lake. The river is 710 km long, and its 71,600 km^2^ drainage basin is characterized by intense land use for agriculture, livestock, energy generation, and urban supply (FEPAM 2010).

The middle course of the Jacuí River is located in the transition zone between the physiogeographic regions of the lower Northeast Slope and Central Depression, with altitudes from 50 to 500 meters above sea level (Pereira et al. 1989). The climate is humid subtropical (Cfa, according to Köppen's classification) with warm and rainy summers, but considered temperate locally (Maluf 2000). Rainfall is regularly distributed throughout the year, with mean precipitation varying from 1500 to 1708 mm, and the monthly mean temperature ranges from 13° C during winter to 18–22° C during summer (Pereira et al. 1989; Maluf 2000). The native vegetation is mainly Seasonal Semideciduous Forest (*sensu* Prado 2000), currently vastly altered, and represented only by small portions of secondary forest, sparsely distributed along rivers and slopes (Longhi et al. 1982; Marchiori et al. 1982).

In 2000, the middle course of the Jacuí River was dammed in its final section by the construction of the Dona Francisca Hydroelectric Power Station (29° 26′ 50″ S; 53° 16′ 50″ W). The water reservoir inundated six municipalities, covering an area of 1337 ha, which contributed to the environmental changes in the region.

### Sampling methods

**Figure 1.**
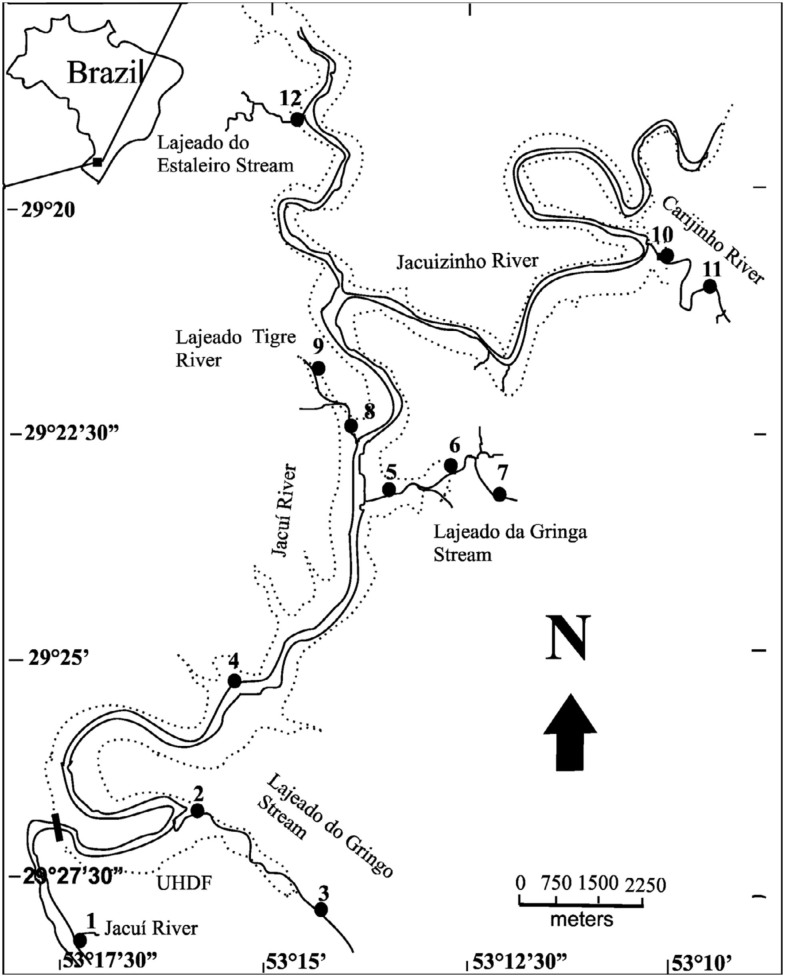
Location of the study area and of sampling sites of the Chironomidae larvae assemblages in the middle course of the Jacuí River, Rio Grande do Sul, Brazil. High quality figures are available online.

Sampling was conducted irregularly between April 2000 and May 2002. Twelve sites were selected for the study, two in the main channel of the Jacuí River, and the remaining sites in its tributaries (seven in three tributaries of the right bank, and three in two tributaries of the left bank) (Figure 1). The environmental characterization of these sites is presented in Table 1. The hydrological classification of stream orders was done on a cartographic scale of 1:50,000, according to Strahler (1957).

The collection was carried out using a Surber-type sampler (area = 0.36 m^2^, mesh = 1 mm). At each site, three subsamples were collected, one in midriver and one near each bank, except in the Jacuí River, where only one of the banks was sampled. The subsamples were pooled in a single sample per site. The sampling took place in shallow water, never deeper than 1 m, in the river. Macrophytes present on the substrate were scraped and collected. The material obtained was fixed in 80% ethanol. The material was sorted, and specimens were counted under a stereoscopic microscope. For taxonomic identification, the specimens were cleared in 10% KOH (potassium hydroxide), mounted on semipermanent slides using Hoyer's medium, and analyzed under an optical microscope (Trivinho-Strixino and Strixino 1995; Epler 2001; Trivinho-Strixino 2011).

Because of the large numbers of specimens in some samples (> 2000), in samples with over 100 larvae, a subsample with 100 specimens was randomly selected for identification purposes. The number of individuals per taxon was estimated afterwards, according to the percentage recorded in the subsample. An ongoing study conducted by the authors, in which all the specimens of chironomids of each sample are identified, showed that there is no difference in the diversity of chironomids in the area studied, if all or only 100 of the specimens are classified. The specimens were identified to genus and/or species level or morphotype using the taxonomic keys of Wiederholm (1983), Trivinho-Strixino and Strixino (1995), Saether et al. (2000), and Epler (2001). Later, the identifications were confirmed by experts.

Voucher specimens were deposited in the Coleção de Zoologia, of the Departamento de Biologia, of the Universidade Federal de Santa Maria, Rio Grande do Sul state, and in the Laboratório de Hidrobiologia of the Universidade Federal de São Carlos, São Paulo state.

### Data analysis

The sampling effectiveness was evaluated by a species accumulation curve (collector curve), which was obtained by the mean of 500 curves generated by the random addition of samples using Estimates 7.5 software (Colwell 2006). This method was chosen because it calculates the fluctuations around the mean curve when the data sets are added, so it is the best method to evaluate the similarity of the inventory to the total richness of the area (Colwell and Coddington 1994).

The composition of the chironomid larvae assemblage recorded in the middle course of the Jacuí River and its tributaries was compared to the assemblages found by inventories conducted at 32 other locations in Brazil (Table 2). Among the inventories found in literature, those with sampling methods favoring benthic fauna were selected. In cases when an area was surveyed more than once, or in cases when several surveys were made in areas close to each other (< 10 km), only the studies with the most complete and/or up-todate lists were included. In the studies carried out in wide areas, such as along the Iguaçu River, in the state of Paraná (Takeda et al. 2005), the taxa list of the assemblage was divided into sectors (i.e., sections of the river course), if the article provided the taxa list by sampling sites. In order to compare the assemblages of different studies, taxa with uncertain identifications were discarded (i.e., Genus A, Genus 1), as well as identifications below genus level, because in many cases such an identification is based on morphotypes (e.g., Suriano and Fonseca-Gessner 2004; Siqueira and Trivinho-Strixino 2005; Rosin et al. 2009), and is not suitable for comparison between different studies.

The similarity between the chironomid larvae assemblages was calculated using the Coefficient of Geographic Resemblance (Duellman 1990). This coefficient is expressed by Coeffecient of Geographic Resemblence = 2Ns/Na+Nb, where Ns = number of species in both areas, Na = number of species in area A. and Nb = number of species in area B. It is equivalent to Sorensen's, Dice's, and Czekanowski's indices (Wolda 1981; Krebs 1999; Magurran 2004), and was performed using NTSYS PC 2.10s software. The matrix of similarity (Coefficient of Geographic resemblence) was later represented by means of clustering analysis with the weighted pair-group method with an arithmetic average (Sokal and Micherner 1958) to avoid the effect of sample size (richness of genera in different assemblages) on the analyses (Valentin 1995). Possible distortions in the graphical representation of the matrix of similarity by clustering analysis were evaluated by means of the cophenetic correlation coefficient (r) (Romesburg 1984). The closer the value of r is to 1, the smaller is the distortion (*sensu* Rohlf 2000).

The similarity among taxa lists of assemblages of inventories can be affected by differences in sampling effort, such as the size of the area surveyed, periodicity of sampling, etc. However, this tendency can be minimized if studies carried out with a low sampling effort on the temporal axis show higher effort on the spatial axis (and vice-versa) (Santos et al. 2009). The differences between these studies are considered here, and reported in Table 2.

Data sets based on a spatially structured schedule may show relationships due to geographical proximity, which is known as spatial autocorrelation (Legendre and Legendre 1998). In order to test this relationship, a matrix of geographical distances between locations was constructed, obtained from the geographical coordinates cited in the publications or found by using Google Earth (http://www.google.com/earth). The correlation between the matrix of geographical distance and the matrix of similarity in the composition of the chironomid larvae assemblages was determined by the Mantel Test (Manly 2000). This test indicates the correlation between matrices based on Z-statistics, where Z depends on the number or magnitude of elements in the matrix to be compared (Valentin 1995). Thus, normalization is needed to transform Z into a coefficient (r) that varies from 1 to -1 (Valentin 2000). The significance of Z was determined by the Monte Carlo permutation test (Smouse et al. 1986), using 5000 permutations. The Mantel and Monte Carlo tests were performed using the NTSYS PC 2.10s software (Rohlf 2000).

**Figure 2.**
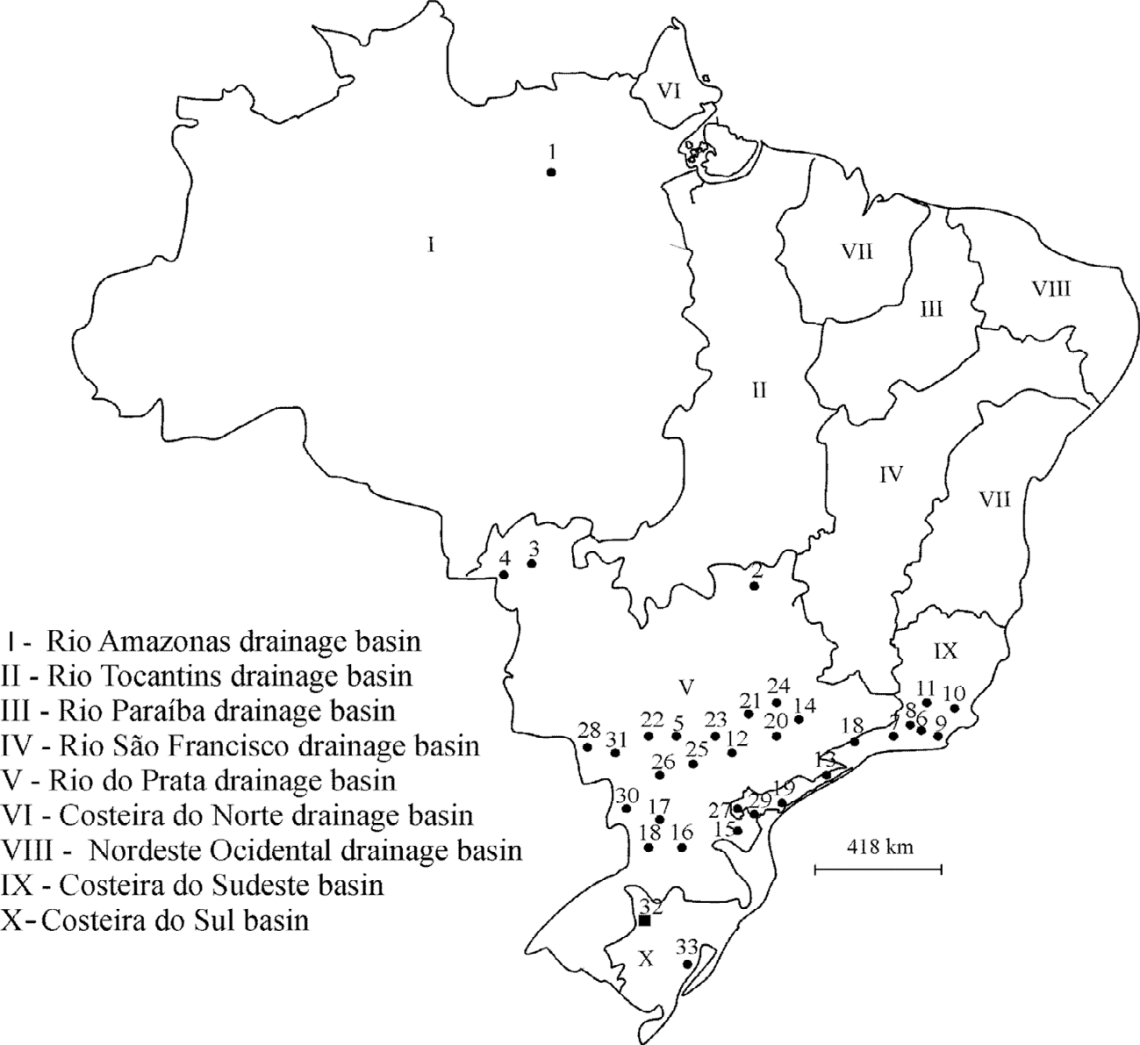
Brazilian hydrographic basins (lnstituto Brasileiro de Geografia e Estatística 2003) and locations (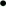) surveyed for the analysis of similarity with the community described in this study (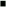), in the middle course of the Jacuí River, Rio Grande do Sul, and those recorded in other Brazilian locations, (see the corresponding number key for each location in Table 1) (Adapted from the website: http://labgeo.blogspot.com/2009/02/mapa-das-bacias-hidrograficas-do-brasil.html). High quality figures are available online.

The observed pattern of the spatial distribution of chironomid larvae assemblages was correlated with four hypothesis matrices, based on regional environmental characteristics designed to explain the pattern that was found:

1) Altitude hypothesis matrix: this postulates that the assemblages of Chironomidae at the locations with similar altitudes are more similar to each other. This hypothesis is based on the altitudes of the different locations of the studies compared (as provided in the reports and/or obtained using the geographical coordinates in the online version of Google Earth). The matrix of similarity of altitude among the locations was obtained through the Euclidean Distance index (Magurran 2004).
2) Biome hypothesis matrix: this postulates that the compositions of the chironomid assemblage at locations in the same biome are more similar to each other than to those of other biomes. This hypothesis matrix is based on the locations of the inventories, and on the Brazilian biome classification (i.e., Cerrado, Amazon Forest, Atlantic Forest, Pantanal, and Pampa, according to the lnstituto Brasileiro de Geografia e Estatística (2003)). A binary matrix was constructed in which pairs of locations from different biomes (i.e., Cerrado and Atlantic Forest) received a similarity value of 0, and pairs of locations from the same biome received a similarity value of 1.
3) Hydraulic conditions (lotic or lentic) hypothesis matrix: this postulates that the chironomid assemblages at locations with the same hydraulic conditions are more similar to each other than to those with different conditions. Reservoirs and other impoundments were considered lentic environments. In order to obtain the matrix of similarity, pairs of locations with different hydraulic conditions received the value 0 (i.e., rivers × lakes), pairs with the same conditions received the value 1, and pairs of locations in which one inventory involved lotic and lentic environments, and another only one of these conditions, received the value 0.5.
4) Hydrographic basin hypothesis matrix: this postulates that the assemblages from locations within the same hydrographic basin are more similar to each other than to those in different hydrographic basins. The hydrographic basin of each study was obtained by plotting (geographical coordinates) the locations on the map of the major hydrographic basins of Brazil. These basins, shown in Figure 2, are: Rio Amazonas (Amazon River), Rio da Prata (Plate River), Costeira Sudeste (Southeast Coastal), and Costeira Sul (South Coastal), according to the lnstituto Brasileiro de Geografia e Estatística (2003). In order to obtain the matrix of similarity, pairs of locations in different hydrographic basins received the value 0, and pairs of locations in the same hydrographic basin received the value 1.

Because of the occurrence of spatial autocorrelation (r = 0.257; *p* = 0.016), a partial Mantel test (Smouse et al. 1986) was run to test the correlation between the matrix of similarity for the composition of the chironomid larvae assemblages and each of the four hypothesis matrices, using the matrix of geographical distance to avoid the effect of geographical proximity. This test consists of the comparison of two matrices (A and B), removing the effects of a third matrix (C, in the present study, corresponds to the geographical distance matrix) on the first two, using a regression of C on A and B. Thus, the residual matrix obtained represents the variations of matrices A and B that cannot be explained by matrix C (Smouse et al. 1986).

Then, the two residual matrices can be compared freely. For similarity and grouping analysis, Mantel and partial Mantel tests were performed using NTSYSpc 2.10S software (Rohlf 2000).

## Results

### Taxonomic composition and richness in the middle course of the Jacuí River

In total, 12,346 larvae of Chironomidae were collected, classified in 99 taxa (84 genera and/or species and 15 morphospecies), attributed to three subfamilies (Table 3). The subfamily that showed the greatest richness was Chironominae (68 taxa). Other subfamilies showed lower richness (Tanypodinae, 11 taxa; Orthocladiinae, 20 taxa). The dominant taxa were *Rheotanytarsus* sp. 1 (18.3%), *Cricotopus* sp. 2 (16.3%), *Cricotopus* sp. 1 (11.2%), *Polypedilum* (*Polypedilum*) sp. 1 (7.2%), *Rheotanytarsus* sp. 2 (7%), *Pseudochironomus* (6.6%), and *Polypedilum* (*Polypedilum*) sp. 2 (6.4%). Of these, only *Rheotanytarsus* sp. 1 occurred at all sites. Twenty-two taxa were rare (up to three larvae), which corresponds to 22% of the total of taxa, and each taxon occurred at only one site (Table 3).

**Figure 3.**
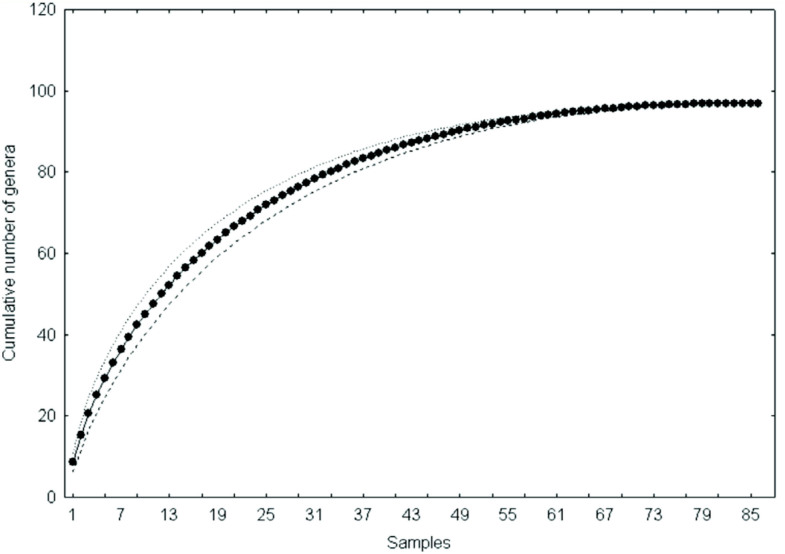
Cumulative curve of taxa of the Chironomidae larvae assemblages recorded in the middle course of the Jacuí River basin, Rio Grande do Sul, Brazil, between April 2000 and May 2002. The solid line with dots represents the mean curve, and the dotted lines represent the variation around the mean. High quality figures are available online.

The species accumulation curve for the 12 sampling sites (88 samples in total) in the middle course of the Jacuí River basin was stable, with little variation along the mean curve, showing that the asymptote was reached (Figure 3).

### Regional distribution pattern

The cluster analysis of the locations with inventories of chironomid larvae assemblages in Brazil showed the formation of five groups with similarity higher than 50%. Group (i) clustered mainly assemblages of locations in the Rio da Prata basin and the Cerrado and Atlantic Forest biomes, although some locations within the Costeira Sul basin and the Pantanal biome were also included (BPCR1, BPCR6, BPCR5, BPMT1, BPCR2, BPCR7, BPMT14, BPMT13, BCSMT, and BPPAN2). Group (ii) clustered mainly assemblages of locations in the Rio da Prata basin and the Atlantic Forest biome, but also included some locations in the Costeira do Sudeste basin and the Cerrado biome (BCSeMT3, BPMT5, BPMT6, BPCR3, BPMT7, BPMT8, BCSeMT7, BPMT12, and BPMT11). Group (Hi) clustered assemblages of locations exclusively in the Atlantic Forest and, except for one location, the Costeira do Sudeste basin (BCSeMT1, BCSeMT5, BCSeMT4, BCSeMT6, BCSeMT2, and BPMT3). Group (iv) clustered locations in two different basins and biomes (BAAM and BCSPM). Group (v) clustered two locations in the Rio da Prata basin and the Atlantic Forest biome (BPMT2 and BPMT10).

The main taxa responsible for clustering the five groups of chironomid larvae assemblages are presented in Table 4. In group i, 21 taxa were frequent, i.e., occurred in 70% or more of the locations compared, such as *Beardius, Caladomyia*, Endotribelos, *Goeldichironomus, Stenochironomus, Clinotanypus, Coelotanypus*, and *Procladius;* furthermore, 11 taxa did not occur in group ii. Group ii contained nine frequent taxa, which were also frequent in locations of group i, as well as taxa that were frequent only within group ii, such as *Democritus, Cladopelma, Dicrotendipes*, and *Fissimentum*. In group iii, 12 taxa were frequent, of which five were not frequent in groups i and ii (*Nimbocera, Oukurriella, Pelomus, Stempellinella*, and *Parametriocnemus*), and five were exclusive (*Nilotanypus, Thienemanniella, Mesosmitia, Pseudosmittia*, and *Rheocricotopus*). Group iv shared the genera *Chironomus, Ablabesmyia*, and *Larsia*, and contained one exclusive taxon (*Macropelopia*). In group ‘v’, the genera *Axarus, Chironomus, Cryptochironomus, Glyptotendipes, Nimbocera, Tanytarsus*, and *Djalmabatista* were shared, and *Glyptotendipes* and *Micropsectra* were exclusive.

The partial Mantel tests (i.e., without spatial autocorrelation effect) indicated weak positive correlations between the distribution of the chironomid larvae assemblages and the hydraulic condition matrix (lotic or lentic), and the matrix of their hydrographic basins (r = 0.153, *p* = 0.05; r = 0.149, *p* = 0.05 respectively). The correlation between the assemblage distribution and the biome matrix was weakly negative (r = -0.136, *p* = 0.05). On the other hand, altitude did not show a significant correlation with the distribution of the chironomid larvae assemblages (r = 0.005, *p* = 0.454).

## Discussion

### Taxonomic composition and richness in the middle course of the Jacuí River

The stability and asymptote reached by the collection curve of the present study suggest that little or no increase would be expected with greater sampling effort. Hence, considering that the occurrence of over 100 species of Chironomidae, most of them rare, in a single river is common (Coffman 1995; Cranston 1995a; Roque et al. 2007), and that a high level of endemism is expected for different biogeographical regions (Coffman 1989), it is possible that many of the 99 taxa found in the middle course of the Jacuí River, of which 20% were rare, are new species. However, as the identification of larvae of Chironomidae at species level is related to the prior description of adults and their associated larvae (Oliver 1971; Pinder 1986; Raunio 2008), it is not possible to tell how many of these taxa might represent new species.

Fourteen genera found in the study area are new occurrences for Rio Grande do Sul (*Aedokritus, Axarus, Endotribelos, Kiefferulus, Manoa, Oukuriella, Phaenopsectra, Stenochironomus, Xenochironomus, Xestochironomus, Cardiocladius, Metriocnemus, Paracladius*, and *Rheocricotopus*). Adding the 99 taxa recorded here to the 13 other genera recorded by Wiedenbrug et al. (1997) and Panatta et al. (2007) for the lakes and wetlands of the Coastal Plain, and by Hepp et al. (2008) and Wiedenbrug et al. (2009) for small streams (*Apedilo, Clinotanypus, Coelotanypus, Onconeura, Alotanypus*, prox. *Macropelopia*, prox. *Adenopelopia, Procladius, Monopelopia, Psectrocladius, Fitkauimya, Paralauterborniella*, and *Stempellina*), the total richness of Chironomidae in Rio Grande do Sul reaches at least 112 taxa.

In terms of geographical patterns, the richness of Chironomidae tends to be greater and to show higher endemism rates in tropical and subtropical climates (Fittkau 1971; Coffman 1989; Cranston 1995b). However, inventories conducted in Brazilian rivers have recorded lower richness levels of genera or species (between 11 and 71) in both tropical (Callisto and Esteves 1998; Marques et al. 1999; Sanseverino and Nessimian 2001; Amorim et al. 2004; Suriano and Fonseca-Gessner 2004; Siqueira and Trivinho-Strixino 2005; Aburaya and Callil 2007; Corbi and Trivinho-Strixino 2008a, b; Silva et al. 2008; Siqueira et al. 2009; Simião-Ferreira et al. 2009; Siqueira et al. 2009) and subtropical (Takeda et al. 2005; Resende end Takeda 2007; Rosin et al. 2009; Sonoda et al. 2009; Rosin et al. 2009) climates than in the cooler temperate climate of this study. Some workers have recorded high richness (200 species) of Chironomidae in temperate lotic environments (e.g., Raunio 2008, in rivers of Finland). Therefore, it is possible that lotic ecosystems constitute an exception to the tendency for richness to be highest in tropical areas, although the available data are somewhat contradictory (McKie et al. 2005). However, other environmental factors can favor high richness of Chironomidae in rivers.

Environmental gradients have been related to the diversity of non-biting midges (Pinder 1995; McKie et al. 2005). The distribution patterns of chironomid subfamilies vary according to the relief. The Orthocladiinae are common in streams located on plateaus, because they are more adapted to cool and well oxygenated waters, while the Chironominae are well adapted to live in lowlands, dwelling in fine sediments, and are tolerant of high temperatures and variations in oxygen content (Pinder 1995). Thus, in regions of transition from rithral to potamic areas, some species live near their limits of ecological tolerance (Statzner and Higler 1986), and so these species can overlap in their distributions (Principe et al. 2008), allowing more species to coexist. The area studied here is a slope, between the upper course (in the uplands of the Planalto) and the lower course (in the lowlands of the Central Depression) of the Jacuí River. Hence, the high richness observed in the middle course of the Jacuí River could be related to its transitional relief.

Environmental heterogeneity is another factor that may promote high richness of macroinvertebrates (e.g., Beisel et al. 2000; Voelz and McArthur 2000; Lencioni and Rossaro 2005). Structurally complex substrates (wood, leaves, stones and gravels, macrophytes, and moss) can provide more niches, with refuges and food resources, as well as protection from predation (Principe and Corigliano 2006), than structurally simple ones (sand or mud). This condition can also facilitate the colonization of middle and lower courses of rivers by taxa characteristic of rithral areas (Tokeshi and Pinder 1985). Many sites of the middle course of the Jacuí River have gravels with encrusting aquatic macrophytes (i.e., *Podostemun*), generating a layer of macrophytes and fine sediments over a gravel substrate. These macrophyte patches increase the heterogeneity of the environment, and probably contributed to the high richness recorded.

The stream order can also influence the composition and richness of chironomid assemblages, especially if the order covaries with altitude, substrate granulometry, and land use (e.g., Lindegaard 1995; Principe et al. 2008; Puntí et al. 2009). In this study, sampling was conducted in stretches from the first to seventh orders. However, altitude (70 to 140 meters above sea level) and granulometry (bolders and cobbles) did not vary among sites, showing no longitudinal gradient. In the middle course of the Jacuí River, the highest richness levels were recorded at four sites (1, 3, 7, 11) ranging from third to seventh order. This result was strongly influenced by the larger sampling effort (higher abundance of larvae) used at those segments (12 samples in each one). In other words, the sampling design does not allow discussion of the role of stream order in affecting the richness of Chironomidae assemblages. The richness levels previously recorded in other Brazilian rivers do not follow a pattern. The highest numbers of species were found in large-order stretches (Takeda et al. 2005; Rosin et al. 2009), but large-order stretches can also show lower richness (Callisto and Esteves 1998; Aburaya and Callil 2007). Thus, climate, relief (slope), and heterogeneity of the substrate possibly play more important roles in affecting the diversity of chironomids in the region studied.

Because sampling in the middle course of the Jacuí River was originally planned to collect mollusks, the abundance and richness of many macroinvertebrates could be underestimated. The mesh size of 1 mm may have allowed many minute specimens to be lost. Thus, the diversity of chironomids might increase if a smaller mesh size were used. However, the sampling area of 0.36 m^2^, and the tangles of *Podostemun* scraped from the stones and added to the samples, in which many chironomid larvae were attached, may have counteracted the large mesh-size effect. In any event, Chironomidae was the most abundant family in the area, and reached the highest richnnes among the insects found in the river (Neri et al. 2005; Spies et al. 2006; Siegloch et al. 2008; Pires 2011).

The predominant taxa in the middle course of the Jacuí River belong to genera that are characteristic of potamic or backwater environments, with stony substrate and litter deposition. *Polypedilum, Rheotanytarsus*, and *Cricotopus* have been recorded in several other Brazilian rivers with stony bottoms (Sanseverino and Nessimian 2001; Roque et al. 2003; Suriano and Fonseca-Gassner 2004) and litter deposition (Sanseverino and Nessimian 2001). *Polypedilum* sp. and *Rheotanytarsus* sp. are characteristic of rapids, with a coarse substrate and turbulent flow (Principe et al. 2008). Even though species of *Polypedilum* are usually associated with fine sediments, some of its species can be found in coarse substrates (Pinder and Reiss 1983).

### Regional distribution pattern

Studies conducted with freshwater macroinvertebrates and other animal groups showed that environmental factors of large spatial scale, such as climate (Bonada et al. 2008), altitude (Maltchik et al. 2010), phytogeographical unit (Santos et al. 2009), and hydrographic basin (Martel et al. 2007), can affect the spatial distribution of the communities. However, the hydraulic conditions of the environments (i.e., lentic or lotic) seem to be the most important factor influencing the macroinvertebrates at the regional scale (Buffagni et al. 2009, 2010). Studies on large-scale patterns of diversity and distribution in lotic environments are few, and are restricted to temperate regions of the Northern Hemisphere (Vinson and Hawkins 1998).

Studies of the regional-scale spatial distribution of chironomid assemblages are also rare. The taxonomic composition of families is more similar in tropical and subtropical areas (Cranston and Naumann 1991; Pinder 1995; Rossaro et al. 2006). The influence of other factors, such as temperature, altitude, phytogeographic unit, hydrography, etc., is little investigated, and the few data available are incipient and/or contradictory. Changes in chironomid assemblages were observed along altitude gradients in European lakes (Bitusík and Svitok 2006), but not in rivers of the bioregion of the Humid Tropics in Australia (McKie et al. 2005). Changes were also observed according to the mean July air temperature in Finland lakes (Luoto 2009). However, chironomid genera have been used to identify the hydraulic conditions of ancient aquatic environments in paleoecological studies (Porinchu and MacDonald 2003), because lotic settings usually support a greater diversity of larvae (Lindergaard 1995; Pinder 1995).

In the present study, the absence of influence of altitude on the spatial pattern of the assemblages, as well as the small degree of influence shown by hydrographic basins, biomes, and hydraulic conditions, probably indicate the relatively small influence of regional environmental factors on the distribution patterns of lotic chironomids. McKie et al. (2005) suggested that the Chironomidae have such a wide tolerance to many environmental variables that they are not affected by regional-scale factors. Additionally, studies conducted with communities of macroinvertebrates and focusing on multiple scales have demonstrated that a large part of the variation in community structure may be influenced by local factors (Rios and Bailey 2006). Hence, the spatial pattern of distribution of chironomid larvae observed in this study may have been more influenced by local factors. Previous studies have shown that local factors, such as oxygen, water velocity and temperature, pH, solid material in suspension, phosphorus, sulfate, presence of algae and macrophytes, type of sediment or substrate, calcium and ferrous ions, and electrical conductivity commonly affect the richness, abundance, and/or composition of communities (Ali et al. 2002; Bisthoven et al. 2005; Inoue et al. 2005; Woodcock et al. 2005; Principe et al. 2008; Siqueira et al. 2008; Al-Shami et al. 2010; Luoto 2011).

Among the taxa that were frequent only in group i, the genera *Beardius, Caladomyia, Endotribelus, Goeldichironomus, Stenochironomus, Clinotanypus, Coelotanypus*, and *Procladius*, and some exclusive genera (*Goeldichironomus, Polypedilum* (*Asheum*)) are characteristic of lentic environments associated with litter, higher temperatures, changes in the hydrological regime, and the presence of macrophytes (Epler 2001). Many of the locations where this group was found show these characteristics, such as the Rio Paraná, Rio Ivinhema, and Saracacáand Carnã creeks. The most frequent and exclusive taxa of group ii (*Aedokrytus, Cladopelma, Dicrotendipes, Fissiomentum, Tanytarsus*, and *Ablabesmyia*) are typical of lentic environments and sandy substrates, and are resistant to certain types of environmental degradation, such as the absence of riparian forest (Pinder and Reiss 1983; Epler 2001). Human activities including agriculture, dams, deforestation, erosion and silting in streambeds, and discharge of industrial and domestic wastes are prevalent in group ii locations.

Among the taxa that were frequent only in group Hi or exclusive to it, *Mesosmitia, Pseudosmittia, Rheocricotopus*, and *Thienemanniella* are characteristic of montane rivers and streams with good environmental preservation (Cranston et al. 1997), conditions shown by the locations of this group (Sanseverino and Nessimian 1998; Roque et al. 2007). The most frequent or exclusive taxa of group iv, such as *Nilothauma, Alotanypus, Labrundinia, Larsia*, and *Macropelopia*, are resistant to environmental degradation (Spies and Reis 1996; Epler 2001). These conditions are found in Porto Trombeta creek and the Bela Vista and Ouro streams (Callisto and Esteves 1998; Panatta et al. 2006).

Among the taxa exclusive to and frequent only in group v, *Axarus, Glyptotendipes, Micropsectra*, and *Paracladopelma* are characteristic of littoral or sublittoral environments, in shallow lentic and lotic environments with slow flow, high temperatures, organic-matter concentration, and fine sediment (mud), in mesotrophic and oligotrophic environments (Pinder and Reiss 1983). The two locations where the genera of group v were found had these characteristics (Takeda et al. 1997; Santos and Henry 2001).

The chironomid larvae assemblage of the middle course of the Jacuí River was most similar to the assemblages found in group i locations, and shared certain frequent genera, such as *Rheotanytarsus, Cricotopus, Polypedilum, Rheotanytarsus*, and *Pseudochironomus*. However, the most abundant taxa found in the area studied here, as previously discussed, are characteristic of lotic environments with rocky substrates and litter deposition, and not of lentic environments, which characterize the environmental preferences of the majority of the frequent species of group i.

Furthermore, the Jacuí River harbored several taxa that were exclusive to this group, such as *Kiefferulus, Xestochironomus, Metriocnemus, Monopelopia, Onconeura*, and *Paracladius*. It is necessary to consider that taxa typical of both uplands and lowlands coexist in the transition regions of the middle course of the Jacuí River, and that the similarity analysis considered only the occurrence of genera.

Local factors such as granulometry, presence of macrophytes, and leaf litter might have influenced the groupings that were formed. However, factors associated with the terrestrial environment, which are usually not taken into account in studies on aquatic communities, might also have influenced the spatial distribution of the Chironomidae. The occurrence of adults of this family is influenced primarily by typically terrestrial factors such as humidity, insolation, shade, air temperature, and predation (Armitage et al. 1995; Tokeshi 1995). Furthermore, it is possible that species, even more than genera, might respond better to the factors tested in our study. For instance, larvae of different species of a single genus (such as *Orthocladius, Rheotanytarsus, Thienemanniella*, and *Polypedilum*) have different environmental preferences in relation to granulometry and water velocity (Pinder and Reiss 1983; Pinder 1995; Epler 2001).

### Final considerations

The richness of larvae of Chironomidae recorded in the middle course of the Jacuí River was higher than those recorded in the warmer tropical and subtropical regions of Brazil. This high richness constitutes even more evidence that lotic ecosystems provide a general exception to the tendency of richness of species to be greater in tropical regions (McKie et al. 2005). This study also corroborates two patterns observed in previous studies of the spatial distribution of lotic Chironomidae: 1) the existence of greater diversity in mountain regions, and in transition zones of rithral and potamic areas, and 2) the possibility that environmental regional-scale factors related to altitude and temperature exert little influence on the distribution of Chironomidae. Moreover, it is important to emphasize the need for additional studies, conducted on local and large spatial scales, especially in Brazil, in order to fill the existing lacunae in knowledge, and to understand the spatial distribution patterns recorded.

**Table 1.**
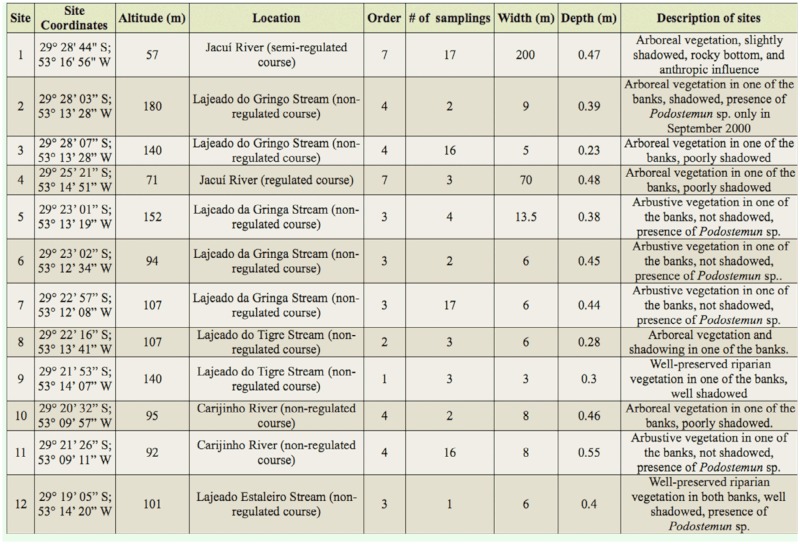
Characterization of the sampling sites of the Chironomidae larvae assemblages collected between April 2000 and May 2002 in the middle course of the Jacuí River, Rio Grande do Sul, Brasil.

**Table 2.**
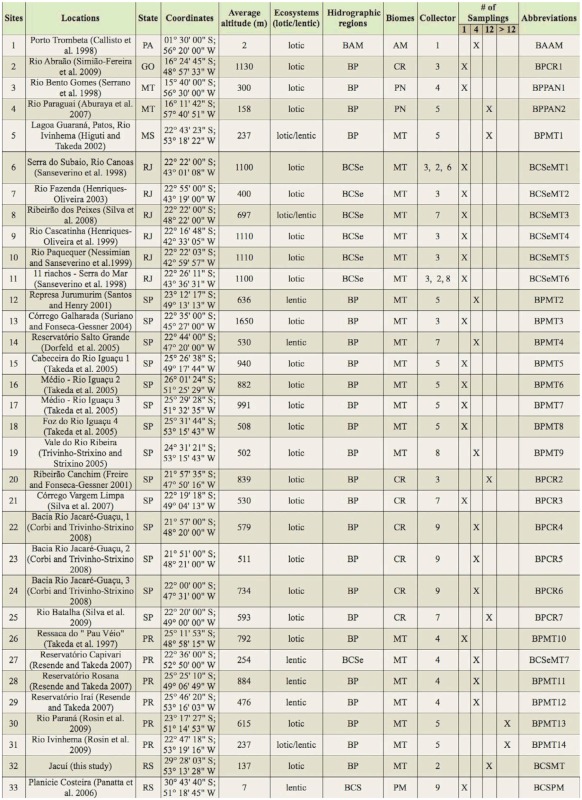
Ecosystems, hydrographic regions, biomes, and respective locations (see references) used in the comparison between the Chironomidae assemblages with those recorded in the middle course of the Jacuí River, Rio Grande do Sul. Hydrographic regions: Bacia Amazônica (Amazon Basin) - AM, Bacia do Prata (Plate River Basin) - BP, Bacia Costeira do Sudeste (Southeast Coastal Basins) - BCSe and Bacia Costeira Sul (South Coastal Basins) - BCS. Biomes: Amazônia - AM, Cerrado - CR, Pantanal - PN, Mata Atlântica - MT and Pampa - PM. Sampler: Core - 1, Manual grab - 2, Surber - 3, Entomological aquatic net - 4, Modified Petersen grab - 5, Manual net - 6, Eckman-Birge grab - 7, D-frame net - 8, Van Veen grab - 9.

Table 3.Taxonomic composition and abundance of the Chironomidae larvae assemblages at 12 sampling sites (1–12) in the middle course of the Jacuí River basin, Rio Grande do Sul, between April 2000 and May 2002. Genera and species identified by capital letters and with Arabic numerals, respectively, correspond to those described in the dichotomouse key of Trivinho-Strixino and Strixino ( 1995), and genera and species identified by Arabic numbers and capital letters, respectively, correspond to those identified by the authors of the present study.

**Table:**
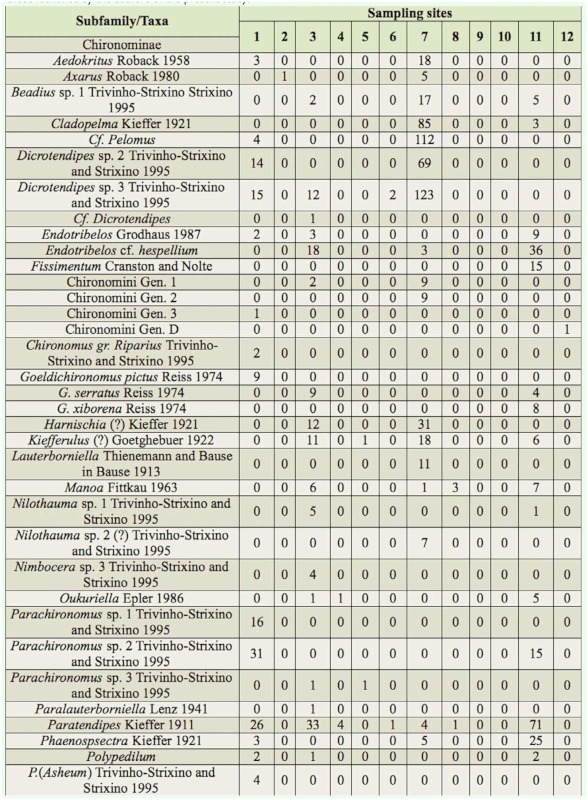

**continued:**
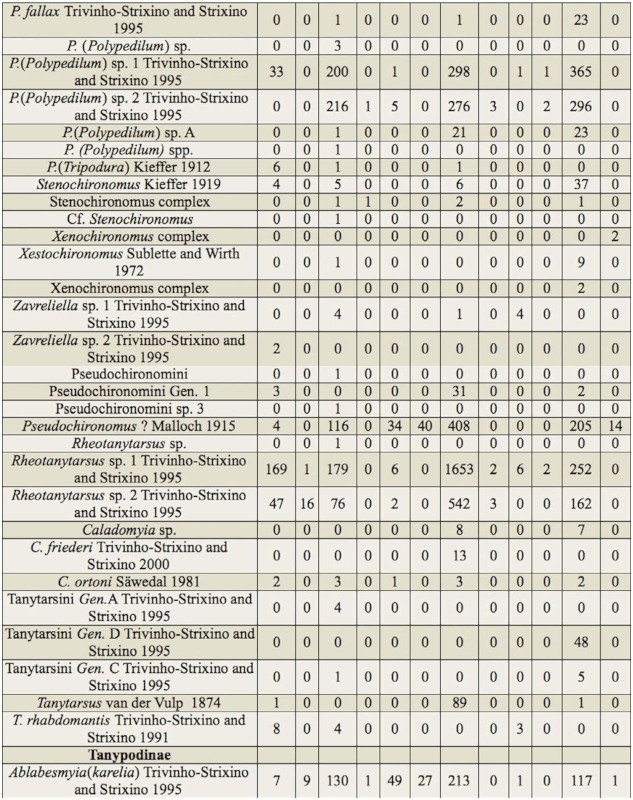

**continued:**
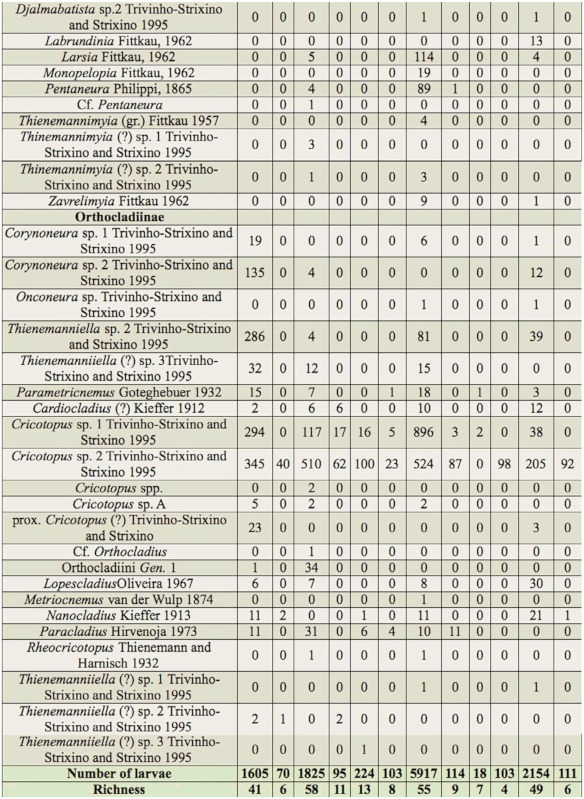

Table 4. Taxonomic composition and frequency of occurrence of the genera of Chironomidae in the five groups formed in the cluster analysis for the 33 locations compared. (Note, i, ii, iii. iv and v = groups formed in the cluster; Arabic numerals in parentheses = number of locations involved in the formation of the groups; bold numbers = taxa with occurrence frequency > 70%; italic numbers = exclusive taxa).

**Table:**
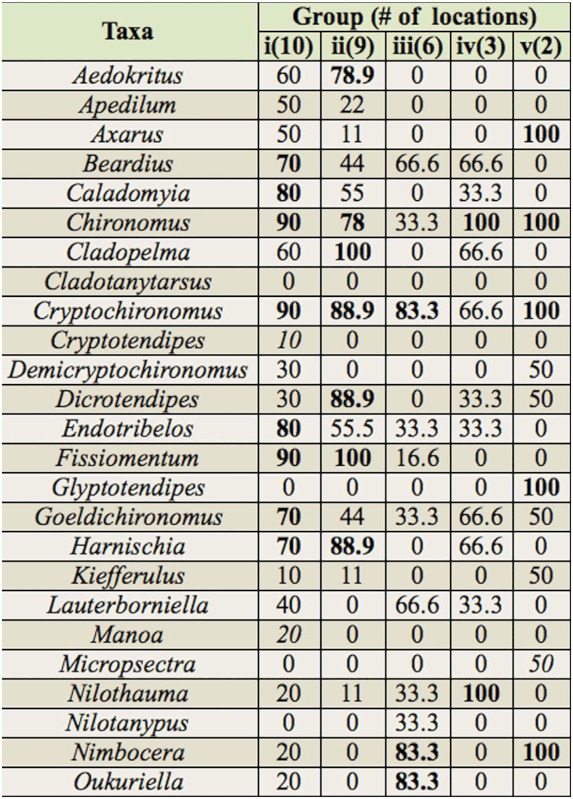

**continued:**
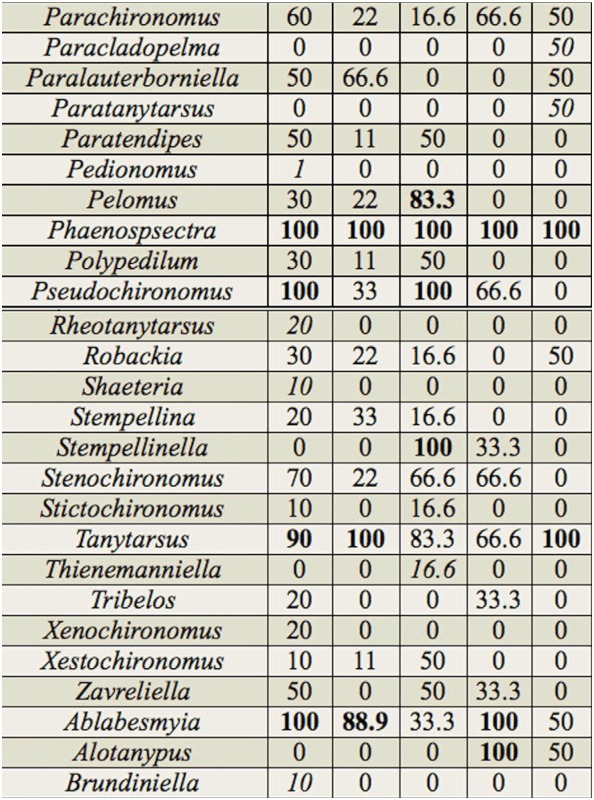

**continued:**
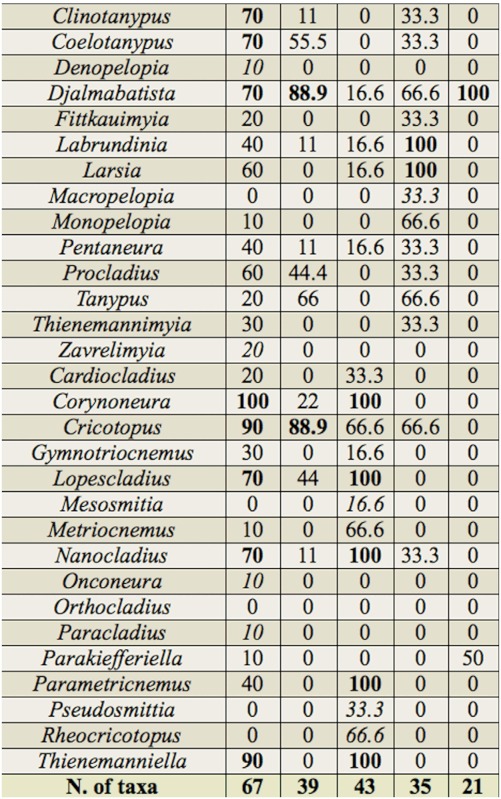

**Figure 4.**
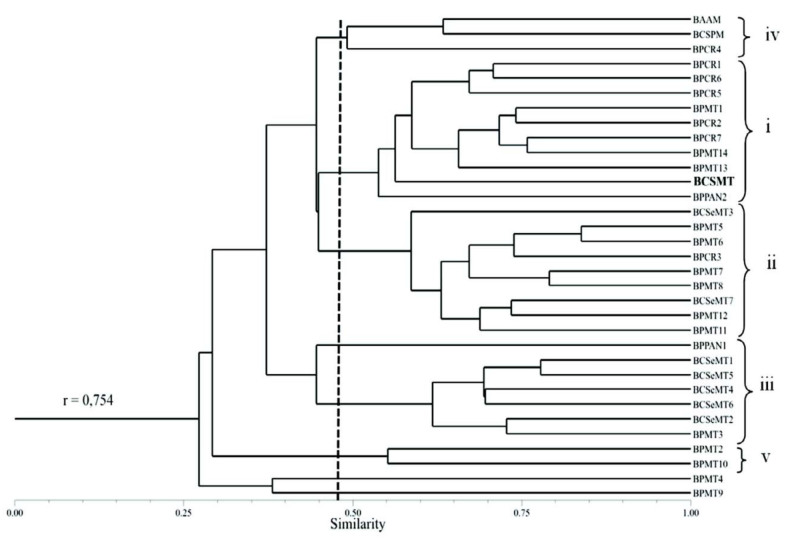
Similarity (Coefficient of Geographic Resemblance, CGR) in the taxonomic composition of the Chironomidae assemblages from inventories taken in Brazil, *r* represents the cophenetic correlation coefficient. The abbreviations for the locations inventoried are defined in Table 2. High quality figures are available online.
